# Perceived social support and Chinese university students’ intentions to participate in non-traditional sports: a moderated chain mediation model

**DOI:** 10.3389/fpsyg.2025.1586099

**Published:** 2025-10-31

**Authors:** Ying Zhang, Mengkai Wang, Yongtao Zhang

**Affiliations:** ^1^Sichuan Tourism University, Chengdu, China; ^2^School of Economics and Management, Chengdu Sport University, Chengdu, China

**Keywords:** perceived social support, psychological resilience, self-efficacy, non-traditional sports, collectivistic orientation

## Abstract

**Aim:**

This study investigates how perceived social support influences Chinese university students’ willingness to participate in non-traditional sports, focusing on the chain mediating roles of psychological resilience and self-efficacy, and the moderating effect of Collectivistic orientation.

**Methods:**

An online survey collected 540 valid responses from Chinese university students. The survey measured perceived social support from family, friends, and significant others; psychological resilience; self-efficacy; and participation intention in non-traditional sports. Main effects and mediation effects were tested using SPSS Process model 6, while moderation effects were examined using Process model 83.

**Results:**

This study revealed that perceived social support positively influences Chinese students’ participation intention in non-traditional sports. Both psychological resilience and self-efficacy partially mediate this relationship, with evidence supporting a sequential mediation pathway. Moreover, the positive effect of perceived social support on psychological resilience is significantly stronger among individuals with a collectivistic orientation, confirming the moderating role.

**Conclusion:**

This study demonstrates that perceived social support significantly enhances participation intention in non-traditional sports among Chinese university students. The chain mediating roles of psychological resilience and self-efficacy, coupled with the moderating influence of cultural orientation, provide valuable insights for promoting non-traditional sports by addressing both psychological and cultural factors.

## 1 Introduction

As a catalyst for youth socialization and cultural innovation, non-traditional sports, such as parkour, skateboarding, and urban climbing, have emerged as global phenomenon, reshaping recreational behaviors and patterns of community engagement among Generation Z (Thorpe and Wheaton, 2021). Defined by an emphasis on self-expression, digital virality, and a hybridization of athletic performance with subcultural identity, these activities transcend conventional competitive frameworks, instead prioritizing participatory enjoyment and social connectivity (Gilchrist and Wheaton, 2017). For adolescents in particular, engagement in such sports provides not only a channel for physical activity but also a unique arena for identity formation, peer bonding, and psychological empowerment, thereby contributing to their holistic development (Holt et al., 2017; Wheaton, 2013). The proliferation of short-video platforms (such as TikTok, Instagram) has further amplified their visibility, transforming them into cultural symbols of youth autonomy and postmodern lifestyle aspirations (Hutchins et al., 2022). Against the backdrop of intensifying global uncertainties and increasing information fragmentation, the de-institutionalized, informal, and diverse characteristics of non-traditional sports have allowed them to evolve into important platforms and bridges for communication among young people worldwide. The 25th World Games, held in Chengdu, China in 2025, further showcased and promoted non-traditional sports on the global stage, generating both substantial economic benefits and cultural influence. However, despite their growing sociocultural importance, such sports continue to face institutional neglect and social stigmatization (Knoppers et al., 2024), which significantly undermine their dissemination and recognition. In this context, it becomes especially important to investigate the factors and psychological mechanisms that support public participation in non-traditional sports, thereby providing targeted policy guidance and practical implications.

Perceived social support, a multidimensional construct encompassing emotional affirmation, instrumental aid, and informational guidance from social networks, serves as a critical enabler of sustained engagement in non-traditional sports (Hailey et al., 2023; Li et al., 2023). Existing research has primarily focused on traditional sports, emphasizing how familial encouragement and peer endorsement enhance athletes’ resilience and long-term commitment (Gupta and McCarthy, 2022; Hoye et al., 2015). However, participants in non-traditional sports often belong to marginalized subcultures with limited mainstream acceptance, rendering conventional forms of social support insufficient or even counterproductive (Thorpe, 2017). For instance, parental concerns about safety risks or societal perceptions of “deviant” behavior may paradoxically weaken support networks, exacerbating participants’ psychological strain (Thorpe et al., 2017). This tension underscores the need to reconceptualize social support dynamics through a cultural lens, particularly within collectivist societies where conformity pressures conflict with individualistic ethos of non-traditional sporting practices (Lee et al., 2019).

Theoretical advancements in resilience and self-efficacy frameworks offer promising avenues for decoding this paradox. Psychological resilience, the capacity to adaptively navigate adversity—has been identified as a protective factor that buffers against participation barriers in stigmatized activities (Gupta and McCarthy, 2022). Concurrently, self-efficacy, defined as the belief in one’s ability to execute goal-directed behaviors may mediate the translation of social support into active engagement. Yet, the interplay between these constructs remains unclear within the context of non-traditional sports. While some studies posit a sequential pathway in which resilience fosters self-efficacy (Sisto et al., 2019), others suggest that cultural values may recalibrate this relationships. For instance, collectivist orientations might amplify the role of community-based support in resilience development (Song and Li, 2023). Despite these insights, no research has systematically examined how cultural orientation moderates the dual mediating roles of resilience and self-efficacy, leaving a critical gap in both theoretical understanding and practical application.

To address this gap, this study proposes a moderated chain mediation model, which posits that perceived social support enhances intentions to participate in non-traditional sports through sequential increases in psychological resilience and self-efficacy, with cultural orientation serving as a boundary condition that either amplifies or constrains these pathways. Specifically, collectivist values are hypothesized to strengthen the link between social support and resilience by legitimizing communal resource-sharing, whereas individualist values are expected to reinforce the transition from resilience to self-efficacy by emphasizing personal mastery narratives. This model not only addresses a theoretical gap in culturally informed sports psychology, but also provides practical implications for public health and youth policy by identifying psychological leverage points for intervention.

## 2 Literature review

### 2.1 The definition of non-traditional sports participation

The concept of non-traditional sports has its roots in the late 20th century, when youth subcultures began to resist the rigid institutionalization and hierarchical structures of mainstream sports such as football, basketball, and athletics (Thorpe and Wheaton, 2021). These emerging activities, often practiced in informal spaces such as streets, parks, and urban environments, were initially labeled as “alternative sports” or “lifestyle sports,” reflecting their countercultural orientation and emphasis on individual freedom (Buning and Walker, 2016). Engaging in physical activity is an effective means for improving overall health (Hu et al., 2025), Non-Traditional Sports belong to Sports activities, but there have been relatively few related studies before. Therefore, this study focuses on Non-Traditional sports. Explore the influence mechanism of Chinese University Students’ Intentions to Participate in Non-Traditional Sports. Subsequent scholarship further elaborated their nature: Cohen et al. (2012) highlighted the centrality of self-expression and cultural identity, while Woods (2021) emphasized their ties to digital media and transnational youth culture. More recent studies have adopted terms such as “new sports” or “non-traditional sports,” underscoring their divergence from standardized, competitive, and rule-bound sport systems (Diez-Fernández et al., 2024; Säfvenbom et al., 2023). Drawing on these definitions, this study conceptualizes non-traditional sports as athletic practices that diverge from mainstream institutional frameworks, prioritize creativity, autonomy, and community interaction, and often integrate subcultural or digital-cultural elements into their practice.

Building on these conceptualizations, several distinctive features of non-traditional sports can be identified. First, they are typically de-institutionalized, with less formal regulation, organizational oversight, or standardized competition compared to traditional sports (Pappous and Hayday, 2016). Second, they emphasize participatory enjoyment, risk-taking, and performative creativity, often blurring the boundary between play, sport, and art (McEwan et al., 2019). Third, non-traditional sports are strongly mediated by digital platforms, where visibility, sharing, and community recognition often outweigh formal achievement or competition outcomes (Woods, 2021). Fourth, their marginal social status means they frequently face stigmatization or neglect, whether due to safety concerns, lack of institutional support, or perceptions of deviance (Darvin et al., 2021). Finally, they possess a global yet locally adaptive nature, functioning both as transnational cultural phenomena and as context-specific practices shaped by local youth identities and urban environments (Skille, 2005). These characteristics differentiate non-traditional sports from traditional institutionalized sports and provide a unique context for examining adolescents’ willingness to participate.

### 2.2 Perceived social support and non-traditional sports participation

Perceived social support (PSS) refers to an individual’s subjective evaluation of the availability and adequacy of emotional, informational, and instrumental support (Lakey and Cassady, 1990). PSS is widely recognized as a central factor in encouraging individuals to engage in physical activity. Its multidimensional conceptualization provides a framework for understanding how resources embedded in social networks facilitate participation (Lakey, 2010). Typically, perceived support derives from three main sources—family, friends, and significant others. In the context of youth sport, encouragement from parents, companionship from peers, and recognition from important social ties can create an environment that reduces uncertainty and enhances participation willingness (Calvete and Connor-Smith, 2006).

Empirical evidence further supports the positive effects of perceived social support on sports involvement. The stress-buffering hypothesis posits that the positive effects of social support are most evident when individuals encounter significant stress (Chen et al., 2021). Within this framework, perceived social support functions as a protective cushion that mitigates the adverse impact of stressors on psychological well-being and behavioral outcomes (Bekiros et al., 2022). This mechanism is particularly relevant in demanding environments, such as competitive sports, where the assurance of available aid can reshape stress appraisals, enhance coping strategies, and sustain engagement. Empirical research supports this view. For example, Rees and Hardy (2004) demonstrated that perceived social support effectively buffers the negative effects of stress, providing evidence consistent with the stress-buffering hypothesis. Moreover, the type of social support provider, as well as the quality and quantity of support significantly influence an individual’s level of sports participation. Although many studies have examined the role of social support in traditional competitive sports, its influence in the context of non-traditional sports remains insufficiently investigated. Given the unique challenges posed by novelty and stigma, social support from family, peers, and significant others may be particularly critical in alleviating apprehension and motivating youth to engage in such activities.

Based on the above discussion, the reviewed literature suggests that although non-traditional sports possess distinctive characteristics and face unique social barriers, perceived social support may positively influence young people’s willingness to participate. Therefore, this study proposes the following hypothesis:

*H1*: Perceived social support positively influences young people’s willingness to participate in non-traditional sports.

### 2.3 The mediating role of psychological resilience

The protective factor framework (Wolfowicz et al., 2021) provides a robust theoretical foundation for examining the mechanisms that underlie youth participation in sports (Hill et al., 2021). This framework emphasizes that individuals’ adaptation to adversity depends on the dynamic interplay between external protective resources (e.g., family, peers, social support) and internal protective resources (such as psychological resilience, self-regulation, coping strategies). When external resources are available, they can activate and strengthen internal psychological resources, which in turn promote positive adaptation (Gupta and McCarthy, 2022; Purcell et al., 2022).

Within this perspective, psychological resilience functions as an internal mechanism that enables individuals to reinterpret difficulties, regulate negative emotions, and persist in goal-directed behaviors. Psychological resilience is typically defined as the ability to maintain or quickly restore functioning following exposure to stress or adversity (Sisto et al., 2019; Vella and Pai, 2019). It represents a process of positive adaptation that involves cognitive appraisal, emotional regulation, and behavioral adjustment in the face of challenges (Fletcher and Sarkar, 2013). In some cases, psychological resilience not only enables resistance to negative outcomes but also fosters personal growth and learning (Egan et al., 2024). By reinforcing individuals’ capacity to adapt, it provides a critical psychological resource that sustains long-term engagement in demanding activities. Psychological resilience is widely recognized as a key determinant of sustained sports participation (Cortês Neto et al., 2020; Sheng et al., 2024). In competitive contexts, resilience enables athletes to cope with adversities such as injuries, performance pressure, and setbacks by fostering adaptability, effective stress management, and a strong “bounce-back” capacity that supports continued engagement (Bryan et al., 2019; Gupta and McCarthy, 2022). Moreover, interventions aimed at enhancing resilience—such as peer mentoring or mental health literacy programs—have been shown to increase participation intentions, underscoring its role as a vital psychological resource for promoting active lifestyles (Mira et al., 2023).

However, most existing research has centered on professional and competitive settings (Durand-Bush et al., 2023; Sarkar and Page, 2022), leaving recreational and non-traditional sports relatively underexplored. According to the protective factor framework, external resources like perceived social support are particularly effective when they reinforce internal protective factors that enable individuals to cope with challenges and achieve positive adaptation (Wolfowicz et al., 2021). In the case of non-traditional sports, where participants often face novelty, safety concerns, and social stigma, psychological resilience represents a decisive internal resource (Woods, 2021). It allows young people to reinterpret these barriers as manageable and even motivating, thereby transforming external support into concrete behavioral intentions. In this sense, resilience functions as the psychological pathway through which perceived social support translates into stronger willingness to participate in non-traditional sports.

Based on this reasoning, the following hypothesis is proposed:

*H2*: Psychological resilience mediates the influence of perceived social support on adolescents’ willingness to participate in non-traditional sports.

### 2.4 The mediating role of self-efficacy

Within the framework of Social Cognitive Theory (Bandura, 1986), self-efficacy is regarded as a central mechanism through which external environments influence individuals’ motivation and behavior. The theory emphasizes reciprocal determinism among personal factors, behavior, and the environment (Marcionetti and Castelli, 2023), suggesting that supportive social contexts can strengthen individuals’ beliefs in their ability to successfully perform specific tasks, thereby enhancing their likelihood of action (Chan, 2022). This perspective provides a theoretical basis for understanding how perceived social support may foster adolescents’ willingness to participate in non-traditional sports through the development of self-efficacy (Ma et al., 2023).

Self-efficacy, defined as an individual’s belief in their capacity to execute behaviors necessary for required to achieve specific goals (Bandura and Wessels, 1997), is a critical determinant of sports participation motivation (Taylor et al., 2025). In the context of non-traditional sports, which often require adaptability and creativity due to their unconventional nature, self-efficacy serves as a key psychological resource (Wei et al., 2025). Individuals with high self-efficacy are more likely to view challenges as manageable and to engage in new or demanding physical activities with greater confidence, consequently, it enhances the willingness to experiment with less structured and stigmatized activities such as non-traditional sports. Meanwhile, from the perspective of Social Cognitive Theory, self-efficacy, as a typical personal factor, develops under the influence of external social support. Existing research has widely confirmed that perceived social support, as an environmental factor (Jaureguizar et al., 2024), can significantly enhance individuals’ self-efficacy (Wang Y. et al., 2024). Within this theoretical framework, strengthened self-efficacy may, in turn, further promote adolescents’ willingness to participate in non-traditional sports as a behavioral factor.

Based on the above discussion, we propose:

*H3*: Self-efficacy mediates the relationship between perceived social support and participation intention in non-traditional sports.

### 2.5 The chain mediating role of psychological resilience and self-efficacy

The conservation of resources (COR) theory (Hobfoll and Shirom, 2001) posits that individuals strive to obtain, retain, and protect valuable resources, and the presence of external resources—such as perceived social support—can generate positive resource gain spirals. In this process, external social resources are converted into internal psychological resources, which subsequently reinforce one another to foster adaptive outcomes. The COR theory provides a useful framework for understanding the potential chain-mediating effects of psychological resilience and self-efficacy (Chen et al., 2024; Tu et al., 2021).

While prior sections have examined the mediating effects of psychological resilience and self-efficacy, emerging research suggests that these two constructs may also operate in a sequential, chain-mediated manner. Specifically, perceived social support may first enhance psychological resilience, which subsequently strengthens self-efficacy (Bingöl et al., 2019; Liu et al., 2018). From a COR perspective, the emotional stability and adaptive coping capacity provided by resilience represent an initial resource gain, which further contributes to individuals’ confidence in their competencies—self-efficacy—as a secondary resource (Lin et al., 2020; Warshawski, 2022). This cumulative process ultimately fosters stronger intentions to participate in non-traditional sports (Dinh and Bonner, 2023).

Empirical evidence supports this sequential mechanism. In the sports domain, individuals with higher psychological resilience are better equipped to confront setbacks and persist in the face of adversity (Sheng et al., 2024; Zhang et al., 2024). As resilience enables athletes to recover and adapt effectively, their sense of mastery and control over challenging tasks increases, thereby reinforcing self-efficacy. This cascading pathway is particularly relevant in non-traditional sports, which often involve social stigma or deviation from conventional norms. For young people—who are especially sensitive to both external support and internal coping resources—this chain mediation may play an instrumental role in shaping their willingness to engage in such activities.

Based on this rationale, the following hypothesis is proposed: H4: Psychological resilience and self-efficacy sequentially mediate the relationship between perceived social support and young people’s intentions to participate in non-traditional sports.

### 2.6 The moderating role of collectivistic orientation

Collectivistic orientation refers to the degree to which individuals define themselves in relation to others and prioritize group goals over individual aspirations (Li et al., 2024). Among those with a strong collectivistic orientation—whether shaped by cultural background or personal values—there is greater emphasis on interpersonal harmony, shared responsibility, and mutual support (Zhang and Han, 2023). This orientation not only enhances the perceived value of social support but also integrates it more deeply into individuals’ psychological coping processes (Özcan and Bulus, 2022; Wu et al., 2011).

Previous research has shown that individuals with collectivistic orientation are more likely to seek out and value external support, viewing it as essential to well-being and stress management (Taylor et al., 2004; Zhang and Han, 2023). The cognitive and emotional frameworks of collectivistic individuals enable a deeper internalization of social support, which contributes to enhance psychological resilience. In the face of adversity, they are more inclined to rely on their social networks, facilitating more adaptive responses (Miller et al., 2017). By contrast, individuals with an individualistic orientation may rely more heavily on personal coping strategies and self-reliance, which could reduce the impact of social support on resilience (Buse et al., 2013). Given these differences, it is reasonable to expect that the impact of perceived social support on psychological resilience varies depending on cultural orientation. Specifically, individuals with a collectivistic orientation are more likely to derive psychological resilience from social support than their individualistic counterparts.

Based on the above, this study proposes the following H5: The positive effect of perceived social support on psychological resilience is stronger for individuals with a collectivistic orientation than for those with an individualistic orientation.

## 3 Research design

### 3.1 Research participants

This study used the ‘Wenjuanxing’ platform to create and generate an online questionnaire link, which was distributed through social media platforms such as Weibo and WeChat. All respondents were informed in advance that the questionnaire would be used solely for academic research purposes. A total of 614 questionnaires were distributed, targeting Chinese university students. Participants in the survey have been informed of the relevant guidelines and have provided their informed consent to participate in the questionnaire, adhering to the ethical standards of the research. After screening the responses, 540 valid questionnaires were collected, resulting in an effective response rate of 87.95%. The sample characteristics are as follows: 311 valid responses were from females (57.6%) and 229 from males (42.4%). Age distribution: 371 respondents were aged 18–25 (68.7%), and 169 were aged 26–30 (31.3%). In terms of education level, 158 respondents had an associate degree (29.3%), 261 had a bachelor’s degree (48.3%), and 121 had a master’s degree or higher (22.4%).

In terms of geographical distribution, 42.5% of participants were from eastern provinces (e.g., Beijing, Shanghai, Jiangsu, and Zhejiang), 29.3% from central regions (including Hubei, Anhui, Henan, and Hunan), and 28.2% from western provinces (such as Sichuan, Chongqing, Yunnan, and Gansu). Regarding the city-tier classification of participants’ hometowns, 29.4% were from first-tier cities (e.g., Beijing, Shanghai, Chengdu), 37.8% from second-tier cities (e.g., Zhengzhou, Changsha, Suzhou), and 32.8% from third-tier cities or rural counties.

### 3.2 Instruments

In this study, the independent, mediating, moderating, and dependent variables were all measured using a seven-point Likert scale for subsequent model validation and hypothesis testing. On this scale, a higher score indicates a greater degree of perception or agreement from the respondent regarding the variable being measured.

Specifically, for the independent variable, a higher score represents stronger perceived social support in the respondent’s daily life. For the two mediating variables, higher scores indicate greater psychological resilience and self-efficacy. Regarding the moderating variable, a higher score signifies a stronger collectivist orientation. Finally, for the dependent variable, a higher score reflects a greater willingness to participate in sports. Conversely, lower scores on the scale correspond to lower levels of perception or agreement with these respective variables.

### 3.3 Scale selection and compilation

Since the original instruments were developed in English, we conducted a standard translation and back-translation procedure to ensure linguistic and cultural validity for the Chinese context. The translated items were reviewed by bilingual experts, and minor adjustments were made based on pilot feedback to improve clarity and conceptual equivalence.

#### 3.3.1 Social support scale

Perceived social support was measured using the scale developed by Vaux, Phillips, and Holly (Vaux et al., 1986), which is divided into three parts: family support, friend support, and other support, comprising 12 items in total. It uses a 7-point Likert scale ranging from 1 (strongly disagree) to 7 (strongly agree), with higher scores indicating greater social support. The Cronbach’s alpha for this scale is 0.714.

#### 3.3.2 Psychological resilience scale

This study employed a simplified version of the Connor-Davidson Resilience Scale, adapted for the Chinese cultural context (Yu and Zhang, 2007). This scale consists of 7 items and uses a 7-point Likert scale ranging from 1 (strongly disagree) to 7 (strongly agree). Higher scores indicate higher levels of psychological resilience. The Cronbach’s alpha for this scale is 0.921.

#### 3.3.3 Self-efficacy scale

This study utilized a simplified version of the Self-Efficacy Scale developed by Schwarzer and Jerusalem (1995). to measure self-efficacy. The scale comprises 10 items across three dimensions and employs a 7-point Likert scale (1 = strongly disagree; 7 = strongly agree), with higher scores indicating stronger self-efficacy among participants. The Cronbach’s alpha for this scale is 0.927.

#### 3.3.4 Collectivistic orientation scale

This study employed the cultural orientation scale developed by Bierbrauer et al. (1994), selecting 16 items specifically focused on collectivistic orientation. Participants rated each item on a 7-point Likert scale (1 = strongly disagree; 7 = strongly agree), with higher scores indicating a stronger collectivistic orientation. The Cronbach’s alpha for this scale is 0.953.

#### 3.3.5 Project participation willingness scale

**Figure 1:**
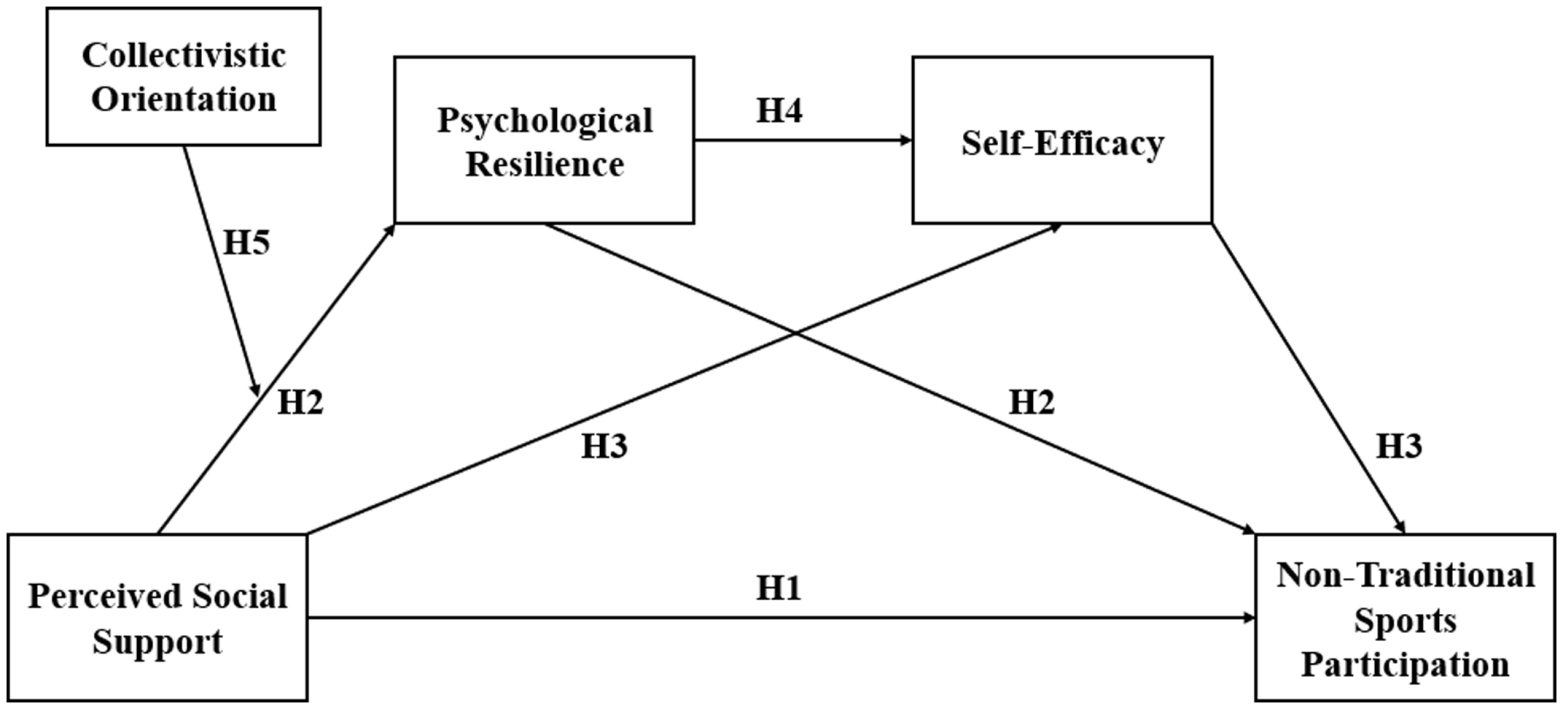
Conceptual model.

The subscale of project participation willingness contains 3 items. The sources of the scale items are summarized based on existing research (Elliott et al., 2003). The Likert seven-point scale is adopted, where 1 to 7 represents “strongly disagree” to “strongly agree.” In practical applications, respondents can express their degree of agreement or disagreement with each item on this scale. The overall reliability of this scale is 0.859, which indicates a relatively high level of internal consistency and stability (Figure 1).

## 4 Results

Reliability analysis was conducted using SPSS 26.0 on the scales employed in this study. Excluding demographic information items, the overall internal consistency reliability of the scale was found to be 0.924. The Cronbach’s alpha values for each subscale were as follows: perceived social support (*α* = 0.714), psychological resilience (*α* = 0.921), self-efficacy (*α* = 0.927), collectivistic orientation (*α* = 0.953), and project participation willingness (*α* = 0.859).

To examine the construct validity of the scales, a Kaiser–Meyer–Olkin (KMO) test and Bartlett’s test of sphericity were conducted on the 48 items included in the scales. The KMO value was 0.892, and Bartlett’s test was significant (*p* < 0.01), indicating that the data were suitable for factor analysis (Table 1).

**Table 1:** Reliability test results of the scale.

Latent variable	Latent variable	Cronbach’s *α*
Total scale	48	0.914
Perceived social support	12	0.714
Psychological resilience	7	0.921
Self-efficacy	10	0.927
Collectivistic orientation	16	0.953
Project participation willingness	3	0.859

### 4.1 Common method bias test

Given that the data in this study were collected via self-report questionnaires, it is necessary to test for common method bias, as recommended by Podsakoff et al. (2003). The Harman single-factor test was employed to examine common method bias. Excluding demographic items, factor analysis was conducted on the study variables using SPSS 26.0. Principal component analysis with eigenvalues greater than 1 was used to extract factors, followed by varimax (orthogonal) rotation. An exploratory factor analysis of the 48 measurement items revealed that the first factor accounted for 24.411% of the total variance, which is well below the commonly accepted threshold of 40%. These results suggest that common method bias is not a serious concern in this study. Detailed results are presented in Table 2.

**Table 2:** Results of KMO and Bartlett spherical test.

KMO and Bartlett test		Value
KMO value		0.892
Bartlett	Approximate chi-square	23305.150
	df	1,128
	P	<0.001

### 4.2 Correlation analysis

Using SPSS 26.0, a correlation analysis was conducted to examine the relationships among the independent variables, mediating variables, and dependent variables across their respective dimensions. As shown in Table 3, all correlations were significant at the 0.05 level (two-tailed), indicating meaningful associations among the variables. These results provide a sound basis for the subsequent analyses in this study.

**Table 3:** Correlation matrix and discriminant validity test.

	M	SD	PSS	PR	S-E	CO	PPW
CR			0.971	0.936	0.938	0.967	0.802
AVE			0.738	0.678	0.603	0.746	0.576
Variables							
PSS	4.425	0.561	**0.867**				
PR	4.462	1.141	0.227**	**0.771**			
S-E	3.656	0.964	0.195**	0.324**	**0.774**		
CO	4.340	1.001	0.172**	0.158**	0.120**	**0.797**	
PPW	4.462	0.966	0.322**	0.576**	0.306**	0.160**	**0.778**

**Correlation is significant at the 0.01 level (two-tailed); *Correlation is significant at the 0.05 level (two-tailed).

PSS refers to perceived social support; PR refers to psychological resilience; S-E refers to self-efficacy, CO refers to Collectivistic Orientation, PPW refers to project participation willingness.

The bold values represent the square roots of the AVE values.

### 4.3 Mediating effect test

The mediating effect test in this study was conducted using the PROCESS macro in SPSS, employing the bias-corrected percentile Bootstrap method with 5,000 resamples. A chained mediation model was constructed, with perceived social support as the independent variable, participation intention in non-traditional sports as the dependent variable, and psychological resilience and self-efficacy as mediating variables. The results are presented in Table 4. Perceived social support had a significant positive effect on participation intention (*β* = 0.576, *t* = 8.131, *p* < 0.001), psychological resilience (*β* = 0.438, *t* = 5.565, *p* < 0.001), and self-efficacy (*β* = 0.230, *t* = 3.178, *p* < 0.05). When all three variables—perceived social support, psychological resilience, and self-efficacy—were included in the regression model simultaneously, each had a significant positive effect on participation intention: perceived social support (*β* = 0.337, *t* = 5.497, *p* < 0.001), psychological resilience (*β* = 0.461, *t* = 13.604, *p* < 0.001), and self-efficacy (*β* = 0.107, *t* = 2.943, *p* < 0.05).

**Table 4:** Mediating effect model test.

	PR	SE	PPW	PPW
Constant	2.520** (7.168)	1.425** (4.335)	2.309** (7.290)	0.922** (3.279)
PSS	0.438** (5.565)	0.230** (3.178)	0.576** (8.131)	0.337** (5.497)
PR		0.238** (5.317)		0.461** (13.604)
SE				0.107** (2.943)
*R* ^2^	0.054	0.122	0.109	0.383
Adjusted *R*^2^	0.053	0.118	0.108	0.379
*F*	*F*(1,538) = 30.966, *p* = 0.000	*F*(2,537) = 37.161, *p* = 0.000	*F*(1,538) = 66.108, *p* = 0.000	*F*(3,536) = 100.714, *p* = 0.000

*p < 0.05, **p < 0.01.

Further analysis of the mediation pathways (see Table 5) revealed that the 95% confidence interval (CI) for the total indirect effect of psychological resilience and self-efficacy did not include zero, indicating a significant chained mediation effect (indirect effect = 0.16), accounting for 47.06% of the total effect. Perceived social support influenced Chinese university students’ intention to participate in non-traditional sports through three significant mediation paths: (1) Perceived social support → psychological resilience → participation intention. The 95% CI of the mediating effect did not contain zero, confirming the significance of this mediation pathway (mediating effect = 0.202, accounting for 35.094% of the total effect). (2) Perceived social support → self-efficacy → participation intention. The 95% CI of the mediating effect did not contain zero, indicating a significant mediation effect (mediating effect = 0.025, accounting for 4.340% of the total effect). (3) Perceived social support → psychological resilience → self-efficacy → participation intention. The 95% CI of the mediating effect did not contain zero, confirming a significant chained mediation effect (mediating effect = 0.013, accounting for 2.257% of the total effect). In conclusion, H1, H2, H3, and H4 were all supported.

**Table 5:** Chain mediating effect analysis of psychological resilience and self-efficacy.

	Effect value	Boot SE	Bootstrap 95%CI		Proportion
			LLCI	ULCI	
Total effect	0.576	0.082	0.437	0.715	100.000%
Direct effect	0.337	0.061	0.217	0.458	58.507%
Total indirect effect	0.239	0.024	0.093	0.185	41.493%
PSS-PR-PPW	0.202	0.022	0.073	0.161	35.094%
PSS-SE-PPW	0.025	0.007	0.004	0.029	4.340%
PSS-PR-SE-PPW	0.012	0.003	0.002	0.014	2.257%

### 4.4 Moderating effect test

A moderated mediation model was subsequently tested. After standardizing and centering the measured variables, PROCESS Model 83 was employed to examine the moderating effect of collectivism orientation in the relationship between perceived social support and psychological resilience. The results (see Table 6) revealed a significant interaction effect between perceived social support and collectivism orientation on psychological resilience among Chinese university students (*β* = 0.171, SE = 0.080, *t* = 2.131, *p* = 0.034). This confirms that collectivism orientation significantly and positively moderated the relationship between perceived social support and psychological resilience.

**Table 6:** Summary of moderating effect analysis.

	PPW	PR	SE
Constant	0.970** (3.459)	5.245** (3.566)	2.483 (1.797)
PSS	0.323** (5.319)	−0.322 (−0.948)	0.177 (0.553)
CO		−0.612 (−1.750)	−0.054 (−0.165)
PSS*CO		0.170* (2.131)	0.032 (0.428)
PR	0.463** (13.653)		
SE	0.108** (2.982)		
*R* ^2^	0.380	0.0874	0.046
Adjusted *R*^2^	0.376	0.067	0.039
*F*	*F*(3,536) = 109.730, *p* = 0.000	*F*(3,536) = 14.259, *p* = 0.000	*F*(3,536) = 8.628, *p* = 0.000

*p < 0.05, **p < 0.01.

Furthermore, we examined the chained mediation effect under different levels of collectivistic orientation (see Table 7). The conditional indirect effect analysis showed that the 95% bootstrap confidence intervals for the two mediators—psychological resilience and self-efficacy, under both high and low levels of collectivistic orientation did not include zero, supporting the validity of the moderated mediation model.

**Table 7:** Conditional indirect effect.

Mediator variables	Standard	Value	Effect	BootSE	BootLLCI	BootULCI
PR	−1SD	3.338	0.114	0.054	0.015	0.226
	Average	4.340	0.193	0.038	0.119	0.270
	+1SD	5.342	0.272	0.058	0.160	0.386
SE	−1SD	3.338	0.031	0.014	0.007	0.060
	Average	4.340	0.034	0.014	0.010	0.066
	+1SD	5.342	0.038	0.019	0.007	0.082

**Figure 2:**
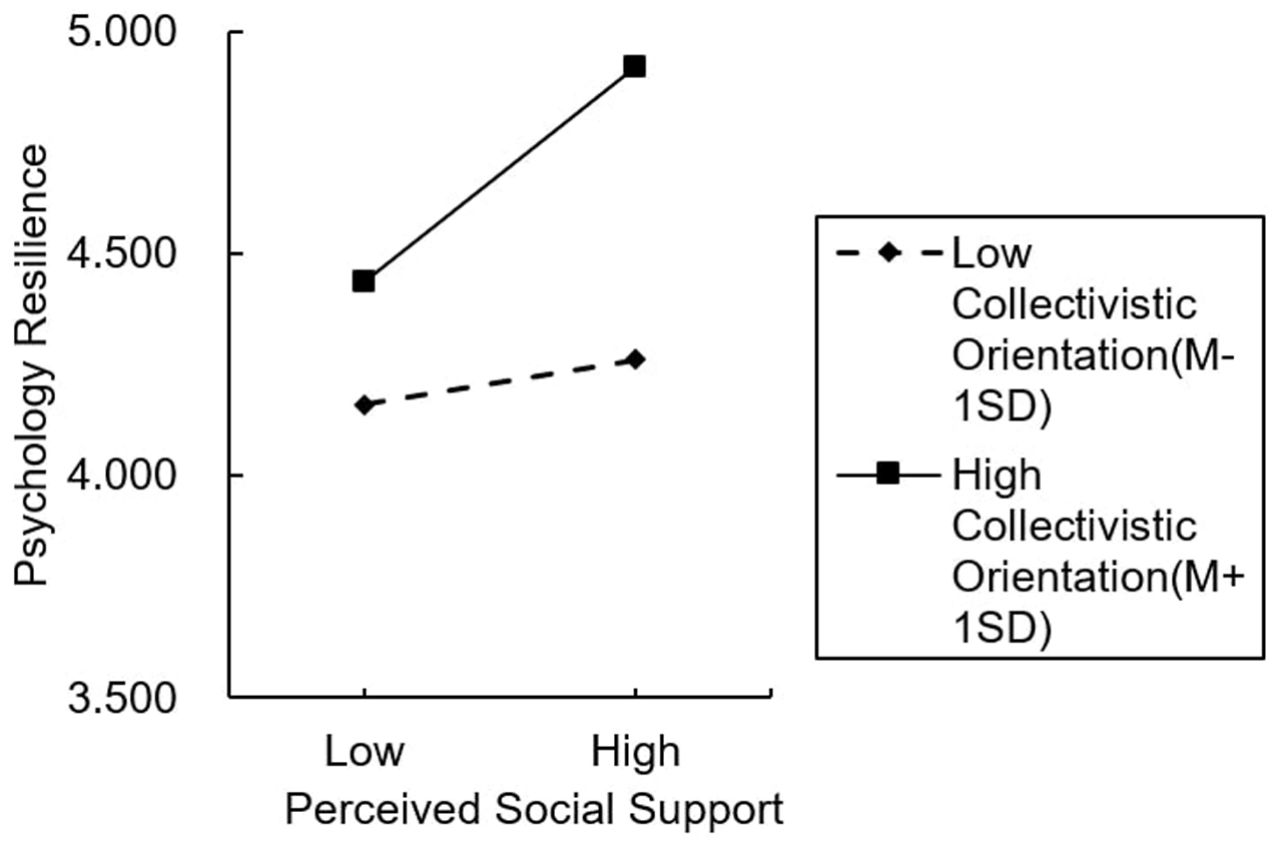
Simple slope graph.

To better illustrate the specific mechanism of this moderating effect, a simple slope analysis was conducted by plotting psychological resilience at ±1 standard deviation of perceived social support and collectivism orientation (see Figure 2). As shown in the figure, compared to students with a low collectivism orientation, those with a high collectivism orientation exhibited a stronger positive effect of perceived social support on their intention to participate in non-traditional sports. Therefore, H5 was supported.

## 5 Discussion

### 5.1 Direct impact

The empirical findings supported the hypothesized direct relationships. Specifically, perceived social support significantly predicted intention to participate in non-traditional sports (H1), and it served as a strong predictor of both psychological resilience (H2) and self-efficacy (H3). These results align with contemporary theoretical frameworks that conceptualize social support as a critical resource in fostering behavioral engagement and promoting psychological adaptation. Notably, recent studies has emphasized that social support exerts an even stronger influence within collectivistic contexts, where relational interdependence amplifies its effects (Lakey and Orehek, 2011).

Moreover, the moderating effect of collectivistic orientation was evident: individuals with stronger collectivistic tendencies benefited more from social support. This finding corroborates cultural psychology research demonstrating that collectivistically oriented individuals, by prioritizing harmony, are more effective at leveraging their social networks to navigate novel or challenging circumstances (Triandis, 2018).

### 5.2 The mediating role of psychological resilience between perceived social support and non-traditional sports participation

Psychological resilience, defined as the ability to effectively cope with and recover from adversity, emerged as a important mediator linking perceived social support to participation intention in non-traditional sports. The results indicated that greater social support not only directly encouraged sports participation but also significantly enhanced psychological resilience, which in turn facilitated higher levels of engagement. This aligns with recent research by Gupta and McCarthy (2022), who emphasized the critical role of resilience in sustaining participation in demanding sports environments. Recent studies have further underscored the buffering capacity of resilience. For instance, Wang S. et al. (2025) found that individuals with higher levels of resilience are more capable of managing stress and uncertainty, enabling continued participation despite personal or social challenges. In our study, psychological resilience appeared to function as an “emotional shield,” transforming adversity into opportunities for growth and sustained involvement.

Moreover, by fostering adaptive coping strategies, resilience enables individuals to reframe setbacks as learning experiences rather than deterrents. Its dual role, as both an outcome of social support and a facilitator of continued engagement, emphasizes the importance of integrating resilience-building interventions into sports participation programs. For example, initiatives that combine stress management workshops, peer mentoring, and community support can significantly enhance the resilience of potential participants, ultimately leading to higher rates of sustained involvement in non-traditional sports.

### 5.3 The mediating role of self-efficiency between perceived social support and non-traditional sports participation

Self-efficacy also emerged as a critical mediator in converting perceived social support into participation intention. Based on social cognitive theory, this study found that verbal affirmations and encouragement from peers, coaches, or family members significantly boosts confidence, particularly when individuals face initial apprehensions. For instance, novice parkour participants often shift from fear to enthusiasm upon receiving positive feedback, a phenomenon similarly observed in studies of esports (Trotter et al., 2022) and adventure tourism (Wang P.-Y. et al., 2024), where mentorship plays a central role in developing self-belief. This enhanced self-efficacy not only motivates individuals to take initiative but also sustains persistent when confronted with setbacks. It transforms doubt into confidence, creating a self-reinforcing cycle in which increased self-efficacy both initiates and maintains participation. These findings highlight the importance of incorporating structured mentorship and positive reinforcement in intervention strategies to foster long-term commitment to non-traditional sports.

### 5.4 The chain mediating role of psychological resilience and self-efficiency between perceived social support and non-traditional sports participation

This study revealed that perceived social support influences participation intention in non-traditional sports through a chain mediation process involving psychological resilience and self-efficacy. Specifically, individuals who perceived high levels of social support initially develop greater psychological resilience, enabling them to effectively cope with the inherent challenges of non-traditional sports. His resilience then contributed to greater self-efficacy, reinforcing belief in one’s capabilities and ultimately increasing intention to participate.

This chain mediation model extends existing theoretical frameworks by demonstrating that the impact of social support unfolds through interconnected psychological mechanisms rather than in isolation. Drawing on Bandura’s social cognitive theory (Bandura, 2001), the findings suggest that initial support experiences reduce stress, enhance resilience, and build confidence, which collectively drive sustained behavioral engagement.

Practically, these insights highlight the importance of designing comprehensive intervention programs that simultaneously enhance social support, resilience, and self-efficacy. For example, initiatives combining emotional encouragement with skill-building activities can effectively cultivate resilience, thereby boosting confidence and increasing participation in non-traditional sports.

### 5.5 The moderating role of collectivistic orientation

The operationalization of collectivistic orientation through culturally adapted items revealed a critical amplification mechanism: individuals with stronger collectivistic tendencies were more effective in translating perceived social support from family, peers, and significant others, into enhanced psychological resilience. This finding aligns with cultural psychology theories asserting that collectivistic cultures prioritize interdependence and facilitate greater utilization of social resources (Li et al., 2024). Specifically, this study expands Li (2022)’s findings by applying this framework to the context of youth sports participation, demonstrating that cultural orientation not only shapes access to social support but also influences its effectiveness in resilience-building.

Our findings resonate with previous work by Zang et al. (2014) and Finkelstein (2011), who reported proactive support-seeking behavior in collectivistic contexts. However, our study advances this literature by clarifying the underlying transformation mechanisms—such as group cohesion and internalization of shared values—that enable collectivistically oriented individuals to convert support into resilience. Practically, the stronger effect sizes among collectivistic individuals suggest the need for culturally embedded intervention strategies. For example, family-based resilience programs or community-led mentoring initiatives may be particularly effective in collectivistic societies, while alternative approaches emphasizing personal goals and autonomy may be more appropriate in individualistic settings.

Based on the findings, schools and community organizations are encouraged to develop structured social support systems—such as peer mentorship programs, inclusive extracurricular activities, and culturally responsive counseling services—that reinforce students’ sense of belonging and competence. These interventions can help foster psychological resilience and strengthen self-efficacy, particularly among youth navigating stigmatized or less institutionally supported forms of sport participation.

## 6 Conclusion

This study provides strong empirical support for the proposed conceptual model linking perceived social support to participation in non-traditional sports. Specifically, perceived social support not only directly predicts participation intention but also serves as a significant antecedent of both psychological resilience and self-efficacy. Furthermore, our findings reveal that psychological resilience and self-efficacy function as both independent and sequential mediators: enhanced resilience leads to greater self-efficacy, which in turn strengthens participation intention. Notably, the moderation analysis demonstrated that a stronger collectivistic orientation amplifies the positive impact of perceived social support on psychological resilience. These integrated results underscore the critical role of supportive social environments and culturally informed approaches in promoting engagement in non-traditional sports. They offer valuable implications for designing targeted interventions that foster long-term involvement through both psychological and sociocultural pathways.

Beyond the immediate empirical findings, this study makes several meaningful contributions. Theoretically, it advances the understanding of culturally embedded psychological mechanisms by integrating perceived social support, psychological resilience, and self-efficacy into a moderated chain mediation model. This framework extends existing theories of motivation and participation into the domain of non-traditional sports and adapts them to collectivist cultural contexts. Practically, the study provides actionable insights for educators, policymakers, and sport promoters—suggesting that interventions targeting psychological resilience and self-efficacy, especially when aligned with collectivistic values, may be more effective in encouraging youth participation in alternative forms of physical activity.

## 7 Limitations and future research

Despite the valuable insights gained, this study has several limitations. Future research should consider expanding the sample scope, adopting longitudinal designs, and examining the findings within the broader Chinese cultural context. Additionally, investigating potential differences across age and gender, as well as applying the frameworks of perceived social support and psychological resilience in other cultural contexts.

Moreover, future studies should also account for regional and urban–rural differences in China, as such contextual factors may shape not only access to non-traditional sports but also public attitudes and the structure of social support networks. Stratified sampling by region and city tier may help further elucidate these macro-level influences.

## Data Availability

The raw data supporting the conclusions of this article will be made available by the authors, without undue reservation.

## References

[ref1] Bandura A. (1986). Social foundations of thought and action: a social cognitive theory. Englewood Cliffs, NJ, US: Prentice-Hall, Inc.

[ref2] Bandura A. (2001). Social cognitive theory: an agentic perspective. Annu. Rev. Psychol. 52, 1–26. doi: 10.1146/ANNUREV.PSYCH.52.1.1 11148297

[ref3] Bandura A. Wessels S. (1997). Self-efficacy: Cambridge University Press Cambridge.

[ref4] Bekiros S. Jahanshahi H. Munoz-Pacheco J. M. (2022). A new buffering theory of social support and psychological stress. PLoS One 17:e0275364. doi: 10.1371/journal.pone.0275364, PMID: 36223401 PMC9555651

[ref5] Bierbrauer G. Meyer H. Wolfradt U. (1994). Measurement of normative and evaluative aspects in individualistic and collectivistic. Theory, method, and applications. eds. U. Kim, H. C. Triandis, Ç. Kâğitçibaşi, S.-C. Choi, and G. Yoon. (pp. 189–199). Sage Publications, Inc.

[ref6] Bingöl T. Y. Batik M. V. Hosoglu R. Firinci Kodaz A. (2019). Psychological resilience and positivity as predictors of self-efficacy. Asian J. Educ. Train. 5, 63–69.

[ref7] Bryan C. O'Shea D. MacIntyre T. (2019). Stressing the relevance of resilience: a systematic review of resilience across the domains of sport and work 12, 70–111. doi: 10.1080/1750984X.2017.1381140

[ref8] Buning R. J. Walker M. (2016). Differentiating mass participant sport event consumers: traditional versus non-traditional events. Sport Mark. Q. 25, 47–58. doi: 10.1177/106169341602500106

[ref9] Buse N. A. Bernacchio C. Burker E. J. (2013). Cultural variation in resilience as a response to traumatic experience. J. Rehabil. 79:15.

[ref10] Calvete E. Connor-Smith J. K. (2006). Perceived social support, coping, and symptoms of distress in American and Spanish students. Anxiety Stress Coping 19, 47–65. doi: 10.1080/10615800500472963

[ref11] Chan R. C. H. (2022). A social cognitive perspective on gender disparities in self-efficacy, interest, and aspirations in science, technology, engineering, and mathematics (STEM): the influence of cultural and gender norms. Int. J. STEM Educ. 9:37. doi: 10.1186/s40594-022-00352-0

[ref12] Chen G. Wang J. Huang Q. Sang L. Yan J. Chen R.. (2024). Social support, psychological capital, multidimensional job burnout, and turnover intention of primary medical staff: a path analysis drawing on conservation of resources theory. Hum. Resour. Health 22:42. doi: 10.1186/s12960-024-00915-y, PMID: 38898452 PMC11186187

[ref13] Chen L. Zilioli S. Jiang Y. Wang X. Lin D. (2021). Perceived social support and children’s physiological responses to stress: an examination of the stress-buffering hypothesis. Biopsych. Sci. Med. 83, 51–61. doi: 10.1097/PSY.0000000000000875 33060454

[ref14] Cohen A. Brown B. Welty Peachey J. (2012). The intersection of pop culture and non-traditional sports: an examination of the niche market of quidditch. Int. J. Sport Manag. Market. 12, 180–197. doi: 10.1504/IJSMM.2012.052666

[ref15] Cortês Neto E. D. Dantas M. M. C. Maia R. d. S. Araújo Filho I. Maia E. M. C. (2020). The resilience of adolescent participants in social projects for sport. Ciênc. Saúde Coletiva 25, 901–908. doi: 10.1590/1413-81232020253.18362018, PMID: 32159660

[ref16] Darvin L. Mumcu C. Pegoraro A. (2021). When virtual spaces meet the limitations of traditional sport: gender stereotyping in NBA2K. Comput. Human Behav. 122:106844. doi: 10.1016/j.chb.2021.106844

[ref17] Diez-Fernández P. Ruibal-Lista B. Revesado-Carballares D. Rodríguez-Cayetano A. López-García S. (2024). Cornerball: a new alternative sport proposal for school physical education. Front. Sports Act. Living 6:1360123. doi: 10.3389/fspor.2024.1360123, PMID: 38803419 PMC11128593

[ref18] Dinh T. T. H. Bonner A. (2023). Exploring the relationships between health literacy, social support, self-efficacy and self-management in adults with multiple chronic diseases. BMC health services research. 23:923. doi: 10.1186/s12913-023-09907-5 37649013 PMC10466814

[ref19] Durand-Bush N. Baker J. van den Berg F. Richard V. Bloom G. A. (2023). The gold medal profile for sport psychology (GMP-SP). J. Appl. Sport Psychol. 35, 547–570. doi: 10.1080/10413200.2022.2055224

[ref20] Egan L. A. Park H. R. Lam J. Gatt J. M. (2024). Resilience to stress and adversity: a narrative review of the role of positive affect. Psychol. Res. Behav. Manag. 17:1403. doi: 10.2147/PRBM.S391403, PMID: 38770188 PMC11104260

[ref21] Elliott M. A. Armitage C. J. Baughan C. J. (2003). Drivers' compliance with speed limits: an application of the theory of planned behavior. J. Appl. Psychol. 88, 964–972. doi: 10.1037/0021-9010.88.5.964, PMID: 14516256

[ref22] Finkelstein M. A. (2011). Correlates of individualism and collectivism: predicting volunteer activity. Soc. Behav. Personal. 39, 597–606. doi: 10.2224/sbp.2011.39.5.597

[ref23] Fletcher D. Sarkar M. (2013). Psychological resilience. Eur. Psychol. 18, 12–23. doi: 10.1027/1016-9040/a000124

[ref24] Gilchrist P. Wheaton B. (2017). The social benefits of informal and lifestyle sports: a research agenda. Int. J. Sport Policy Polit. 9, 1–10. doi: 10.1080/19406940.2017.1293132

[ref25] Gupta S. McCarthy P. J. (2022). The sporting resilience model: a systematic review of resilience in sport performers. Front. Psychol. 13:1003053. doi: 10.3389/fpsyg.2022.1003053, PMID: 36619099 PMC9811683

[ref26] Hailey V. Fisher A. Hamer M. Fancourt D. (2023). Perceived social support and sustained physical activity during the COVID-19 pandemic. Int. J. Behav. Med. 30, 651–662. doi: 10.1007/s12529-022-10125-2, PMID: 36175607 PMC9521870

[ref27] Hill D. M. Brown G. Lambert T.-L. Mackintosh K. Knight C. Gorczynski P. (2021). Factors perceived to affect the wellbeing and mental health of coaches and practitioners working within elite sport. Sport Exerc. Perform. Psychol. 10, 504–518. doi: 10.1037/spy0000263

[ref28] Hobfoll S. E. Shirom A. (2001). “Conservation of resources theory: applications to stress and management in the workplace” in ed. Golembiewski, R. T. Handbook of organizational behavior. 2nd ed (New York, NY, US: Marcel Dekker), 57–80.

[ref29] Holt N. L. Neely K. C. Slater L. G. Camiré M. Côté J. Fraser-Thomas J.. (2017). A grounded theory of positive youth development through sport based on results from a qualitative meta-study. Int. Rev. Sport Exerc. Psychol. 10, 1–49. doi: 10.1080/1750984X.2016.1180704, PMID: 27695511 PMC5020349

[ref30] Hoye R. Nicholson M. Brown K. (2015). Involvement in sport and social connectedness. Int. Rev. Sociol. Sport 50, 3–21. doi: 10.1177/1012690212466076

[ref31] Hu Z. Zhang Y. Liao C. Nong L. Kadier K. Zhu K. (2025). Relationship between physical exercise self-efficacy and persistent exercise behavior among college students. Alpha Psychiatry 26:38955. doi: 10.31083/AP38955, PMID: 40352082 PMC12059754

[ref32] Hutchins B. Rowe D. Ruddock A. (2022). “The commodification and mediatization of fandom: creating executive fandom” in eds. Coombs, D. S. and Osborne, A. C. Routledge handbook of sport fans and fandom (London: Routledge), 365–376.

[ref33] Jaureguizar J. Dosil-Santamaría M. Redondo I. Ozamiz-Etxebarria N. (2024). The mediation of self-esteem and hostility in the relationship between social support and dating violence. Behav. Psychol. 32, 503–521. doi: 10.51668/bp.8324305n

[ref34] Knoppers A. van Doodewaard C. Spaaij R. (2024). (un) doing gender inequalities in sport organizations. J. Sport Manag. 1, 1–9. doi: 10.1123/jsm.2024-0032

[ref35] Lakey B. (2010). Social support: Basic research and new strategies for intervention. New York, NY: The Guilford Press.

[ref36] Lakey B. Cassady P. B. (1990). Cognitive processes in perceived social support. J. Pers. Soc. Psychol. 59, 337–343. doi: 10.1037/0022-3514.59.2.337

[ref37] Lakey B. Orehek E. (2011). Relational regulation theory: a new approach to explain the link between perceived social support and mental health. Psychol. Rev. 118, 482–495. doi: 10.1037/a0023477, PMID: 21534704

[ref38] Lee C. Kim S. Owens M. Liechty T. Kim J. (2019). Engaging with sports related serious leisure and acculturation among Korean graduate students. Ann. Leis. Res. 22, 247–263. doi: 10.1080/11745398.2018.1496463

[ref39] Li W. (2022). Resilience among language learners: the roles of support, self-efficacy, and buoyancy. Front. Psychol. 13:854522. doi: 10.3389/fpsyg.2022.854522, PMID: 35360572 PMC8962401

[ref40] Li K. Wang F. Pi Z. (2024). Culture and self-construal in the age of globalization: an empirical inquiry based on multiple approaches. Front. Psychol. 15:1353898., PMID: 38566949 10.3389/fpsyg.2024.1353898 PMC10985239

[ref41] Li N. Zhao S. Liu C. Dai K. Huang W. (2023). Exploring the relationship between perceived social support and college students’ autonomous fitness behavior: chain mediating effect test. Front. Psychol. 13:1036383. doi: 10.3389/fpsyg.2024.1353898 36817388 PMC9928751

[ref42] Lin M. Wolke D. Schneider S. Margraf J. (2020). Bullying history and mental health in university students: the mediator roles of social support, personal resilience, and self-efficacy. Front. Psych. 10:960. doi: 10.3389/fpsyt.2019.00960, PMID: 31993000 PMC6971115

[ref43] Liu N. Liu S. Yu N. Peng Y. Wen Y. Tang J.. (2018). Correlations among psychological resilience, self-efficacy, and negative emotion in acute myocardial infarction patients after percutaneous coronary intervention. Front. Psych. 9:1. doi: 10.3389/fpsyt.2018.00001, PMID: 29410632 PMC5787139

[ref44] Ma C. M. S. Law M. Y. M. Ma A. M. Y. (2023). “The relationship between social support and physical activity: a moderated mediation model based on the self-determination theory” in ed. Ng, B. Self-determination theory and socioemotional learning (Cham, Switzerland: Springer Nature Switzerland AG), 321–348.

[ref45] Marcionetti J. Castelli L. (2023). The job and life satisfaction of teachers: a social cognitive model integrating teachers’ burnout, self-efficacy, dispositional optimism, and social support. Int. J. Educ. Vocat. Guid. 23, 441–463. doi: 10.1007/s10775-021-09516-w

[ref46] McEwan D. Boudreau P. Curran T. Rhodes R. E. (2019). Personality traits of high-risk sport participants: a meta-analysis. J. Res. Pers. 79, 83–93. doi: 10.1016/j.jrp.2019.02.006

[ref47] Miller J. G. Akiyama H. Kapadia S. (2017). Cultural variation in communal versus exchange norms: implications for social support. J. Pers. Soc. Psychol. 113, 81–94. doi: 10.1037/pspi0000091, PMID: 28240938

[ref48] Mira T. Jacinto M. Costa A. M. Monteiro D. Diz S. Matos R.. (2023). Exploring the relationship between social support, resilience, and subjective well-being in athletes of adapted sport. Front. Psychol. 14:1266654. doi: 10.3389/fpsyg.2023.1266654, PMID: 38144980 PMC10748803

[ref49] Özcan B. Bulus M. (2022). Protective factors associated with academic resilience of adolescents in individualist and collectivist cultures: evidence from PISA 2018 large scale assessment. Curr. Psychol. 41, 1740–1756. doi: 10.1007/s12144-022-02944-z

[ref50] Pappous A. Hayday E. J. (2016). A case study investigating the impact of the London 2012 Olympic and Paralympic games on participation in two non-traditional English sports, judo and fencing. Leis. Stud. 35, 668–684. doi: 10.1080/02614367.2015.1035314

[ref51] Podsakoff P. M. MacKenzie S. B. Lee J.-Y. Podsakoff N. P. (2003). Common method biases in behavioral research: a critical review of the literature and recommended remedies. J. Appl. Psychol. 88, 879–903. doi: 10.1037/0021-9010.88.5.879, PMID: 14516251

[ref52] Purcell R. Pilkington V. Carberry S. Reid D. Gwyther K. Hall K.. (2022). An evidence-informed framework to promote mental wellbeing in elite sport. Front. Psychol. 13:780359. doi: 10.3389/fpsyg.2022.780359, PMID: 35250720 PMC8890033

[ref53] Rees T. Hardy L. (2004). Matching social support with stressors: effects on factors underlying performance in tennis. Psychol. Sport Exerc. 5, 319–337. doi: 10.1016/S1469-0292(03)00018-9

[ref54] Säfvenbom R. Strittmatter A.-M. Bernhardsen G. P. (2023). Developmental outcomes for young people participating in informal and lifestyle sports: a scoping review of the literature, 2000–2020. Soc. Sci. 12:299. doi: 10.3390/socsci12050299

[ref55] Sarkar M. Page A. E. (2022). Developing individual and team resilience in elite sport: research to practice. J. Sport Psychol. Action 13, 40–53. doi: 10.1080/21520704.2020.1861144

[ref56] Schwarzer R. Jerusalem M. (1995). “Generalized self-efficacy scale” in Measures in health psychology: a user’s portfolio. Causal control beliefs. eds. Weinman J. Wright S. Johnston M.. UK: NFER-Nelson. 35–37.

[ref57] Sheng X. Liang K. Li K. Chi X. Fan H. (2024). Association between sports participation and resilience in school-attending students: a cross-sectional study. Front. Psychol. 15:1365310. doi: 10.3389/fpsyg.2024.1365310, PMID: 38725957 PMC11081067

[ref58] Sisto A. Vicinanza F. Campanozzi L. L. Ricci G. Tartaglini D. Tambone V. (2019). Towards a transversal definition of psychological resilience: a literature review. Medicina 55:745. doi: 10.3390/medicina55110745, PMID: 31744109 PMC6915594

[ref59] Skille E. Å. (2005). Individuality or cultural reproduction? Adolescents’ sport participation in Norway: alternative versus conventional sports. Int. Rev. Sociol. Sport 40, 307–320. doi: 10.1177/1012690205060230

[ref60] Song H. Li Z. J. G. N. (2023). Community-based service, psychological resilience and life satisfaction among Chinese older adults: a longitudinal study. Geriatr Nurs 54, 148–154. doi: 10.1016/j.gerinurse.2023.09.004 37788562

[ref61] Taylor S. Martin J. Wilson O. W. Elliot L. Bopp M. (2025). The impact of physical activity enjoyment, exercise self-efficacy, recording physical activity, and exercise goal setting on physical activity levels of college students. Recreat. Sports J. 49, 33–45. doi: 10.1177/15588661241261997

[ref62] Taylor S. E. Sherman D. K. Kim H. S. Jarcho J. Takagi K. Dunagan M. S. (2004). Culture and social support: who seeks it and why? J. Pers. Soc. Psychol. 87, 354–362. doi: 10.1037/0022-3514.87.3.354, PMID: 15382985

[ref63] Thorpe H. (2017). Action sports, social media, and new technologies: towards a research agenda. Commun. Sport 5, 554–578. doi: 10.1177/2167479516638125

[ref64] Thorpe H. Toffoletti K. Bruce T. (2017). Sportswomen and social media: bringing third-wave feminism, postfeminism, and neoliberal feminism into conversation. J. Sports Soc. Issue 41, 359–383. doi: 10.1177/0193723517730808

[ref65] Thorpe H. Wheaton B. (2021). Young Gazan refugees, sport and social media: understanding migration as a process of becoming. Int. Migr. Rev. 55, 902–928. doi: 10.1177/0197918320988247

[ref66] Triandis H. C. (2018). Individualism and collectivism. London, UK: Routledge.

[ref67] Trotter M. G. Coulter T. J. Davis P. A. Poulus D. R. Polman R. (2022). Examining the impact of school esports program participation on student health and psychological development. Front. Psychol. 12:807341. doi: 10.3389/fpsyg.2021.807341, PMID: 35140665 PMC8820392

[ref68] Tu Y. Li D. Wang H.-J. (2021). COVID-19-induced layoff, survivors’ COVID-19-related stress and performance in hospitality industry: the moderating role of social support. Int. J. Hosp. Manag. 95, 102912–102918. doi: 10.1016/j.ijhm.2021.102912, PMID: 35702566 PMC9183452

[ref69] Vaux A. Phillips J. Holly L. Thomson B. Williams D. Stewart D. (1986). The social support appraisals (SS-A) scale: studies of reliability and validity. Am. J. Community Psychol. 14, 195–218. doi: 10.1007/BF00911821

[ref70] Vella S.-L. C. Pai N. B. (2019). A theoretical review of psychological resilience: defining resilience and resilience research over the decades. Arch. Med. Health Sci. 7, 233–239. doi: 10.4103/amhs.amhs_119_19

[ref71] Wang P.-Y. Lyons K. Young T. (2024). Role identities of adventure tour operators in national parks. Curr. Issues Tour. 27, 3017–3029. doi: 10.1080/13683500.2023.2265033

[ref72] Wang Y. Wang L. Yang L. Wang W. (2024). Influence of perceived social support and academic self-efficacy on teacher-student relationships and learning engagement for enhanced didactical outcomes. Sci. Rep. 14:28396. doi: 10.1038/s41598-024-78402-6, PMID: 39551776 PMC11570595

[ref73] Wang S. Wang Y. Zhao L. (2025). Effects of psychological resilience on online learning performance and satisfaction among undergraduates: the mediating role of academic burnout. Asia Pac. Educ. Res., 34, 395–40. doi: 10.1007/s40299-024-00862-1

[ref74] Warshawski S. (2022). Academic self-efficacy, resilience and social support among first-year Israeli nursing students learning in online environments during COVID-19 pandemic. Nurse Educ. Today 110:105267. doi: 10.1016/j.nedt.2022.105267, PMID: 35051871 PMC9719613

[ref75] Wei D. Ren Z. Xue J. Fan Y. (2025). Team vs individual sports in adolescence: gendered mechanisms linking emotion regulation, social support, and self-efficacy to psychological resilience. Front. Psychol. 16:1636707. doi: 10.3389/fpsyg.2025.1636707, PMID: 40949334 PMC12425922

[ref76] Wheaton B. (2013). The cultural politics of lifestyle sports. London, UK: Routledge.

[ref77] Wolfowicz M. Litmanovitz Y. Weisburd D. Hasisi B. (2021). Cognitive and behavioral radicalization: a systematic review of the putative risk and protective factors. Campbell Syst. Rev. 17:e1174. doi: 10.1002/cl2.1174, PMID: 37133261 PMC10121227

[ref78] Woods J. (2021). The associations between traditional and social media and the growth of non-normative sports. Sport Soc. 24, 954–971. doi: 10.1080/17430437.2019.1710132

[ref79] Wu M. S. Yan X. Zhou C. Chen Y. Li J. Zhu Z.. (2011). General belief in a just world and resilience: evidence from a collectivistic culture. Eur. J. Personal. 25, 431–442. doi: 10.1002/per.807

[ref80] Yu X. Zhang J. (2007). Factor analysis and psychometric evaluation of the Connor-Davidson resilience scale (CD-RISC) with Chinese people. Soc. Behav. Pers. 35, 19–30. doi: 10.2224/sbp.2007.35.1.19

[ref81] Zang C. Guida J. Sun Y. Liu H. (2014). Collectivism culture, HIV stigma and social network support in Anhui, China: a path analytic model. AIDS Patient Care STDs 28, 452–458. doi: 10.1089/apc.2014.0015, PMID: 24853730

[ref82] Zhang J. Han T. (2023). Individualism and collectivism orientation and the correlates among Chinese college students. Curr. Psychol. 42, 3811–3821. doi: 10.1007/s12144-021-01735-2

[ref83] Zhang S. Wang L. He Y. Liu J.-D. (2024). The divergent effects of resilience qualities and resilience support in predicting pre-competition anxiety and championship performance. Res. Q. Exerc. Sport 95, 101–109. doi: 10.1080/02701367.2022.2156446, PMID: 36689551

